# Cloning of *Pid2* Homolog from *Oryza officinalis* and Functional Analysis of Rice Blast Resistance in Transgenic Yunjing 37 Lines

**DOI:** 10.3390/plants15081222

**Published:** 2026-04-16

**Authors:** Eman M. Bleih, Lingyun Lei, Jinlu Li, Qiaofang Zhong, Fuyou Yin, Ling Chen, Li Liu, Yun Zhang, Jiaxin Xing, Bo Wang, Cong Jiang, Limei Kui, Dunyu Zhang, Qiaoyun Wang, Zaiquan Cheng, Suqin Xiao

**Affiliations:** 1 Biotechnology and Germplasm Resources Institute, Yunnan Academy of Agricultural Sciences/Yunnan Provincial Key Lab of Agricultural Biotechnology, Kunming 650205, China; 2 Agricultural Research Center, Rice Research and Training Department, Field Crops Research Institute, Kafrelsheikh 33717, Egypt; 3 Institute of Food Crop, Yunnan Academy of Agricultural Sciences, Kunming 650205, China; 4 College of Agriculture and Life Sciences, Kunming University, Kunming 650217, China

**Keywords:** wild rice, *Pid2 homolog*, gene cloning, blast disease, transgenic rice

## Abstract

Rice blast, caused by the fungus *Magnaporthe oryzae*, is one of the most devastating threatening to global rice production. The narrow genetic background of modern rice cultivars exacerbates the shortage of durable resistance resources. In contrast, the wild rice species *Oryza officinalis* harbors abundant stress-resistance alleles and represents a valuable gene pool for identifying novel broad blast-resistance genes. The cloned resistance gene *Pid2* is encoded in a receptor-like protein kinase conferring race-specific resistance against the *M. oryzae* isolate ZB15. In this study, three *Pid2* homologs were isolated from *O. officinalis*. The special allele *Pid2of-MD33* was transformed into “Yunjing 37(YG37), a blast-susceptible japonica rice cultivar” via *Agrobacterium*-mediated transformation. Quantitative real-time PCR analysis showed that *Pid2of-MD33* was consistently expressed in various tissues of *O. officinalis*, with the highest transcript abundance detected in leaf mesophyll cells and plasma membranes. Inoculation with the *M. oryzae* isolate ZB15 revealed that transgenic YG37 lines expressing *Pid2of-MD33* displayed significantly reduced lesion size and pathogen proliferation, suggesting recovered race-specific resistance. These results enrich the resistance gene resources for rice blast research and provide a promising candidate gene for rice blast resistance breeding.

## 1. Introduction

Rice blast, a destructive global rice disease caused by the fungal pathogen *Magnaporthe oryzae*, threatens rice yields and accounts for 10% to 30% of worldwide rice production losses each year [1,2,3]. Currently, while chemical control can partially manage rice blast, it also produces environmental pollution [4,5,6]. Additionally, over-reliance on core parental lines in rice breeding has limited the genetic pool of blast resistance genes in cultivated rice, with many major resistance genes (e.g., *Pi-ta*, *Pi-b*) losing effectiveness due to rapid pathogen population shift.Therefore, discovering new resistance resources, identifying resistance genes, and expanding the genetic diversity of rice parental lines are crucial for germplasm innovation [7,8].

Wild rice species, having undergone long-term natural evolution, harbor a wealth of genes that confer resistance to both biotic and abiotic stresses and retain numerous superior alleles lost in cultivated rice, making them invaluable resources for rice genetic improvement and basic research [9,10]. Wild rice species such as *Oryza. rufipogon* are potential sources of blast resistance genes [11,12], from which the strongly blast-resistant *Pi-ta (+)* allele has been identified. Beyond *O. rufipogon*, other wild rice species like *O. officinalis* also exhibit significant disease resistance potential. For example, the *Pid3* ortholog *Pid3-A4*, which confers substantial resistance to rice blast strains common in Sichuan Province, was identified from *O. rufipogon* [5,13]; *Pi54rh* was discovered in *O. rhizomatis* and provides effective blast resistance. Similarly, *Pi54-of*, a *Pi54* homologous gene cloned from highly blast-resistant *O. officinalis* through allele mining techniques [14], is believed to interact with *Avr-Pi54* through a molecular mechanism different from that of *Pi54*. Additionally, the *Pi69 (t)* gene, derived from *O. glaberrima*, was located in a region containing a cluster of NBS-LRR-like genes, which are potential or promising candidates for *Pi69(t)* [4]. This highlights the importance of exploring alleles from both wild and cultivated rice to better understand genetic variation, evolution, and to identify superior genes.

In recent years, significant progress has been achieved domestically and internationally in mapping rice blast resistance-related genes. To date, over 100 genes and more than 500 quantitative trait loci (QTLs) associated with blast resistance have been identified in rice, with over 40 genes cloned. Most of these genes originated from diverse donor rice varieties or wild rice species [15]. Among the more than 100 identified R genes, 45% derive from japonica varieties, 51% from indica varieties, and the remaining 4% from wild rice, such as *Pi9* isolated from *O. minuta*, *Pi-40(t)* from *O. australiensis*, and *Pirf2-1(t)* from *O. rufipogon*. Notably, over half of the mapped resistance loci cluster on chromosomes 6, 11, and 12. Specifically, at least ten genes, including *Pi2*, *Pi9*, *Piz-t*, and *Pigm*, are located near the 10 Mb physical position on chromosome 6; at least 27 genes or alleles, including those cloned from the *Pik* locus (*Pik*, *Pik-p*, *Pik-m*, *Pik-h*, *Pik-s*, *Pi1*), are located on chromosome 11. At least 22 genes are mapped to chromosome 12 [8,16].

Among the numerous cloned blast resistance genes, Pid2, located near the centromere of chromosome 6, is the only one encoding a receptor-like protein kinase. It is a single-copy gene cloned from the indica rice variety Digu. It encodes a transmembrane receptor-like kinase (RLK) with a coding length of 825 amino acids, which can confer resistance to the *Magnaporthe oryzae* race ZB15 in rice. The amino terminal of this kinase is the extracellular domain, containing a hydrophobic signal peptide, a B-lectin domain and a Plasminogen/Apple/Nematode(PAN) domain, while the carboxy terminal is the intracellular domain with a typical serine/threonine kinase structure. A transmembrane domain (TM) is located between the two domains. The PID2 protein is localized on the plasma membrane, and the difference in the 441st amino acid of its TM domain can distinguish between resistant and susceptible alleles [17,18]. Now, multiple allelic variations of this gene have been identified from various ecotypes of cultivated and wild rice. For example, PID2-Y1B, identified in the indica variety Yixiang 1B, possesses only an H666R substitution. Sequencing analysis of the Pid2 gene in 41 landraces from different countries and regions was realized. A total of 5 accessions of *O. nivara*, and 1 accession of *O. rufipogon* Griff. revealed 9 haplotypes involving differences in 5 amino acid residues. Among them, *Pid2-H3*, *Pid2-H4*, *Pid2-H8*, and *Pid2-H9* share identical amino acid sequences with *Pid2-Y1B*, while *Pid2-H2*, *Pid2-H5*, and *Pid2-H7*, besides the H666R difference, exhibit S321L, F116Y, and R7H amino acid substitutions, respectively. Another study, analyzing sequencing data from 70 indica and 58 japonica varieties, reported that the *Pid2* gene was encoded amino acid sequences acid sequences identical to *Pid2-Y1B* in 49 indica accessions. The function of the Pid2 gene was confirmed via transgenic validation, namely *Pid2-ZS*. Further research indicates that PID2, the unique receptor-like protein kinase, interacts via its kinase domain with E3 ubiquitin ligases such as OsPUB15 and OsPIE3. This forms a complex signaling pathway involving phosphorylation, ubiquitination, subcellular translocation, and proteasomal degradation, collectively regulating the rice immune response against blast strain ZB15. Therefore, screening for *Pid2* allelic variations and manipulating its regulatory pathways (e.g., OsPUB15/OsPIE3) provides important molecular targets and a theoretical basis for breeding rice varieties with broad-spectrum and durable blast resistance [7,18].

*O. officinalis* complex is the largest species group in *Oryza* spp., with more than nine species from four continents, and is a tertiary gene pool that can be exploited in breeding programs for the improvement of cultivated rice [19]. In addition, *Oryza officinalis*, as a wild relative of rice, has developed a rich reservoir of stress-resistance genes through long-term natural selection [20]. It possesses a wealth of genetic diversity for tolerance to biotic and abiotic stresses, being an elite gene pool for the improvement of Asian cultivated rice. Its distant evolutionary relationship and abundance of resistance genes are increasingly positioning it as a strategic resource for breaking bottlenecks in resistance breeding [21,22]. Cloning and characterizing the *pid2* gene from *Oryza officinalis* can help researchers to study its function, regulation, and understand the genetic basis of traits that may be beneficial for rice breeding, such as biotic, abiotic stress tolerance and yield improvement [8,23]. Preliminary identification results from our team showed that *Oryza officinalis* from 10 different populations in Yunnan, including those from Menghai and Jinghong, exhibit varying degrees of resistance to rice blast. Furthermore, the presence of *Pid2* homologous genes was detected in all of them, although their specific sequence information and functions remain unclear. In light of this, this study aims to clone the *Pid2* homologous from *O. officinalis* and conduct an in-depth evaluation of its molecular characteristics and function.

## 2. Results

### 2.1. Genetic Distribution Patterns of the Pid2 Gene in O. officinalis

**Figure 1:**
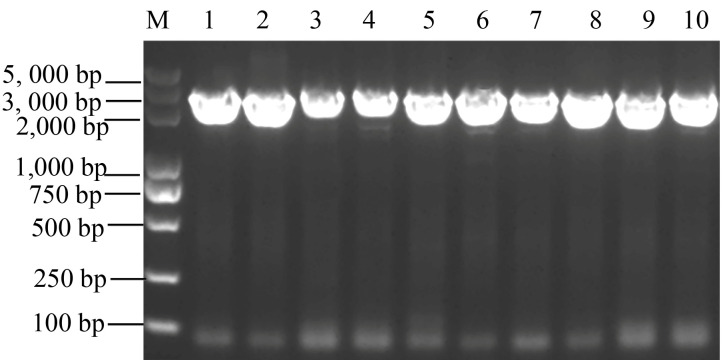
Cloning of *Pid2* homologous gene. M: DL5000 DNA Marker; lanes 1–10: 10 tested accessions of *O. officinalis* materials.

Based on the reference CDS of the *Pid2* gene from *Oryza sativa* indica Digu retrieved from the NCBI database, the distribution of the *Pid2* gene was analyzed in 10 accessions of *O. officinalis* using homologous cloning via primer design and PCR amplification (Figure 1). The results showed that the amplified PCR fragments matched the expected sizes, indicating that homologous *Pid2* genes could be successfully cloned in all 10 tested *O. officinalis* accessions from different populations, suggesting that this gene is widely distributed in *O. officinalis*. The *Pid2* homologous in *O. officinalis* was designated as *Pid2of*.

### 2.2. Sequence Analysis of the Pid2-of Gene in Different Populations of Yunnan O. officinalis

Through sequencing analysis of the *Pid2of* gene in 10 different populations of *O. officinalis* from Yunnan, three genotypes containing the *Pid2of* gene were identified: the Jinghong *O. officinalis* genotype (*Pid2of-JH*), the Mengding28 *O. officinalis* genotype (*Pid2of-MD28*) and the Mengding33 *O. officinalis* genotype (*Pid2of-MD33*). The coding sequence (CDS) region of all three genotypes was 2535 base pairs in length. Between *Pid2of-JH* and *Pid2of-MD28*, only three base pair differences were observed. At the protein level, the three genotypes differed by four amino acid residues. The amino acid sequence similarities between the three *Pid2of* genotypes and the reference *Pid2* gene were as follows: *Pid2of-JH* (97.04%), *Pid2of-MD28* (97.16%), *Pid2of-MD33* (97.05%). Among them, the *Pid2of-MD33* genotype displayed more than 20 amino acid differences compared to the reference PID2 protein. Furthermore, the full-length CDS (2535 bp) of the *Pid2of-MD33* gene shared 95% nucleotide sequence similarity and 97% amino acid sequence similarity with the *Pid2* gene from the Digu strain. The specific base differences among the three genotypes at various loci are detailed in Table 1. Among them, *Pid2of-MD33* was the predominant genotype.

**Figure 2:**
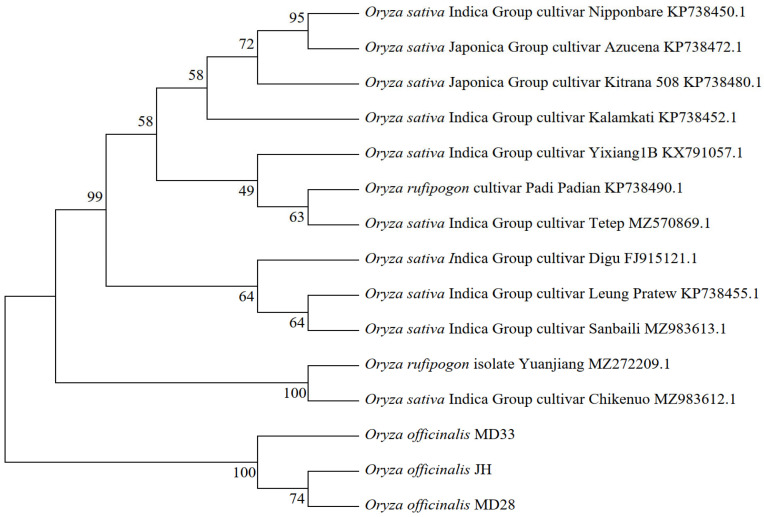
The phylogenetic tree of *Pid2* and its homologous genes.

For phylogenetic tree analysis, The BLAST alignment was performed using the NCBI BLAST online tool (https://blast.ncbi.nlm.nih.gov/Blast.cgi?PROGRAM=blastn&PAGE_TYPE=BlastSearch&LINK_LOC=blasthome accessed on 7 April 2026), and the phylogenetic tree was constructed using MEGA 7.0 software. The analysis revealed that the three homologous genes from *O. officinalis* formed a distinct cluster branch, separate from the alleles found in cultivated rice (*O. sativa*) (Figure 2). The genetic distance among these genes was notably high, indicating significant divergence.

Subsequently, the software DnaSP 5.0 was used to analyze nucleotide polymorphisms among different alleles of *Pid2* in cultivated rice and its homologous gene *Pid2of* in *O. officinalis*. The average number of nucleotide differences (K) was 13.929. The results showed that within the entire CDS region, the PAN domain exhibited the highest values of both nucleotide diversity (π) and Tajima’s D, with a significantly positive Tajima’s D value (*p* < 0.01) compared with other domains. When nucleotide polymorphisms of *Pid2of* in *O. officinalis* were analyzed separately, the PAN domain still showed the highest π value, indicating that the PAN domain of the *Pid2of* gene in *O. officinalis* maintains extremely high sequence diversity at the population level. These results suggest that this region may have been subjected to long-term balancing selection or diversifying selection, enabling it to cope with diverse pathogen pressures within wild rice populations. In contrast, the highly diversified regions of *Pid2* alleles in cultivated rice were concentrated in the PKc-like domain, implying that the PKc-like protein kinase domain of the *Pid2* gene underwent stronger purifying selection during rice domestication and breeding. As the core functional region responsible for disease resistance signal transduction, the sequence conservation of this domain is essential for maintaining the integrity of the defense signaling pathway (Table 2).

### 2.3. Conserved Domain Analysis of the Pid2of-MD33 Genotype

**Figure 3:**
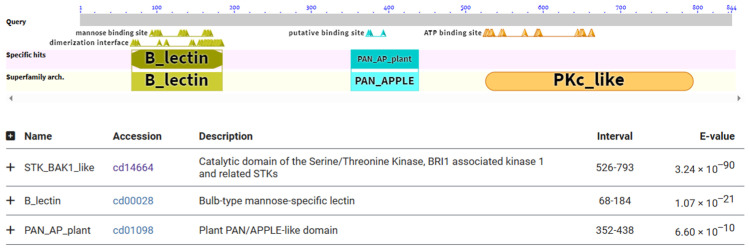
Conserved domains analysis.

Based on previous results indicating that *Pid2of-MD33* is the most dominant genotype among the three analyzed genotypes, subsequent studies have focused on this genotype. The *Pid2of-MD33* gene product was analyzed using the CD-Search tool, revealing that the protein consists of 844 amino acids (aa) and contains several conserved domains. A B-lectin domain is located between aa 68–184, a PAN (plasminogen, apple, nematode) domain spans aa 352–438, and a protein kinase C-like (PKc-like) domain extends from aa 526–793. These structural features suggest potential roles in carbohydrate binding, protein–protein interactions, and signal transduction (Figure 3).

### 2.4. Physicochemical Properties, Secondary Structure Analysis and Tertiary Structure Prediction of Pid2of-MD33 Protein

**Figure 4:**
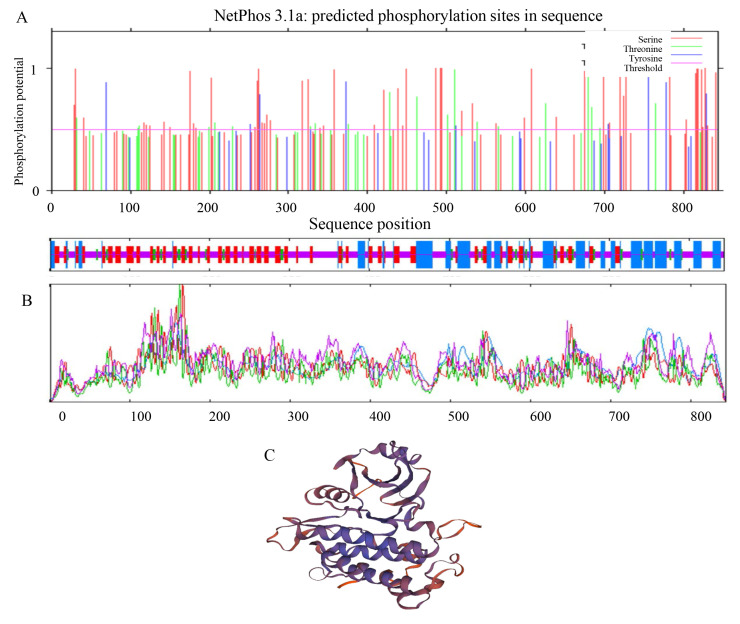
Prediction of phosphorylation sites, the secondary structure and the tertiary structure of PID2OF-MD33. (**A**) Prediction of phosphorylation sites. (**B**) The secondary structure of PID2OF-MD33. (**C**) The tertiary structure of PID2OF-MD33.

A bioinformatics analysis of the *Pid2of-MD33* gene sequence was conducted using ProtParam, revealing several important physicochemical properties of the encoded protein. The molecular formula of the protein is C_4072_H_6302_N_1088_O_1254_S_43_, with a relative molecular weight of 91941.95 Da and a theoretical isoelectric point (pI) of 6.45. The instability index was calculated to be 43.40, indicating that the protein is likely unstable in vitro. The protein comprises 79 positively charged residues (arginine and lysine) and 65 negatively charged residues (aspartic acid and glutamic acid). The aliphatic index was 81.21, suggesting a moderately high thermostability. The GRAVY (Grand Average of Hydropathicity) score was −0.112, indicating that the protein is slightly hydrophilic. Phosphorylation site prediction using the NetPhos 3.1 Server indicated the presence of multiple potential phosphorylation sites, particularly on serine (Ser) and threonine (Thr) residues. These sites suggest that PID2OF-MD33 may undergo post-translational modification, potentially playing a role in regulatory pathways. Secondary structure analysis of PID2OF-MD33 was performed using the SOPMA program. The results indicated the following composition: Alpha helices: 200 residues (23.70%); Beta turns: 50 residues (5.92%); Extended strands (β-sheets): 206 residues (24.41%); Random coils: 388 residues (45.97%). These results suggest that random coils and extended strands are dominant features in the secondary structure of the PID2OF-MD33 protein. Tertiary structure prediction was conducted using the SWISS-MODEL online server. The model predicted that the main structural components of the protein are irregular coils and α-helices, consistent with the secondary structure analysis. These features may be critical to the protein’s functional conformation and interaction with other biomolecules (Figure 4).

### 2.5. Cloning and Functional Characterization of the Pid2of-MD33 Promoter Region

Based on the complete sequence of the *Pid2* allele provided by NCBI, primers were designed to amplify a 2000 bp region upstream of the coding sequence (CDS) of the *Pid2of-MD33* gene in *O. officinalis*. This upstream region was cloned and sequenced, and its regulatory elements were analyzed using the PlantCARE database. The analysis revealed the presence of typical core promoter elements as well as cis-acting elements associated with light responsiveness and light regulation. In addition, several hormone-responsive elements were identified. Notably, the *Pid2of-MD33* promoter contains cis-elements responsive to both biotic stresses such as salicylic acid, jasmonic acid, and gibberellin and abiotic stresses, including drought and aerobic induction. These findings indicated that *Pid2of-MD33* might play a broader role in rice growth and development. Based on the regulatory and functional predictions, it is hypothesized that *Pid2of-MD33* confers a degree of specialized resistance to *M. oryzae* (Table 3).

### 2.6. Predictive Analysis of the PID2OF–MD33 Protein Interaction

**Figure 5:**
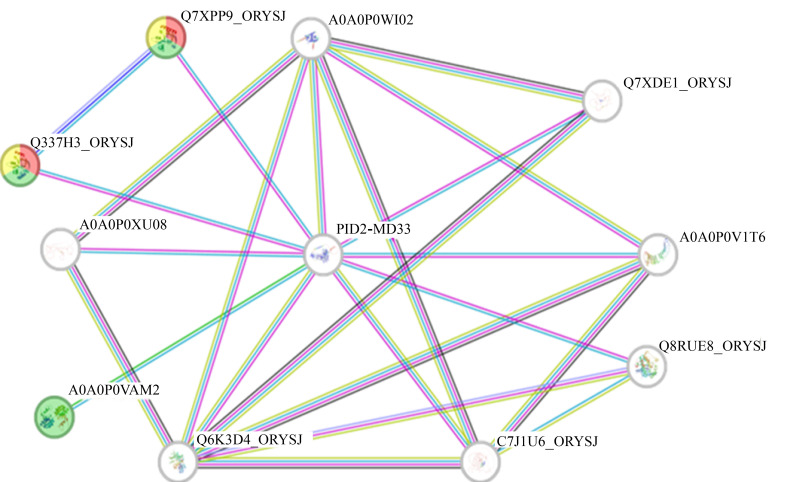
Putative interaction network of PID2OF-MD33.

A protein–protein interaction (PPI) network for *Pid2of-MD33* was constructed using the STRING database to predict potential interacting proteins (Figure 5). The analysis identified 11 nodes in the network, with a clustering coefficient of 0.848 and a PPI enrichment *p*-value of 1, at a confidence level of 0.400. The predicted interacting proteins are primarily associated with 1-acylglycerol-3-phosphoacyltransferase activity and are involved in biological processes such as phosphatidic acid biosynthesis, phospholipid biosynthesis, phosphorus-containing compound metabolism, and E3 ubiquitination. These findings suggested that PID2OF–MD33 may interact with these proteins to regulate rice responses to various biotic and abiotic stresses and may play a broader role in regulating essential rice physiological processes.

### 2.7. Analysis of pid2of-MD33 Gene Expression Across Different Tissues in O. officinalis

**Figure 6:**
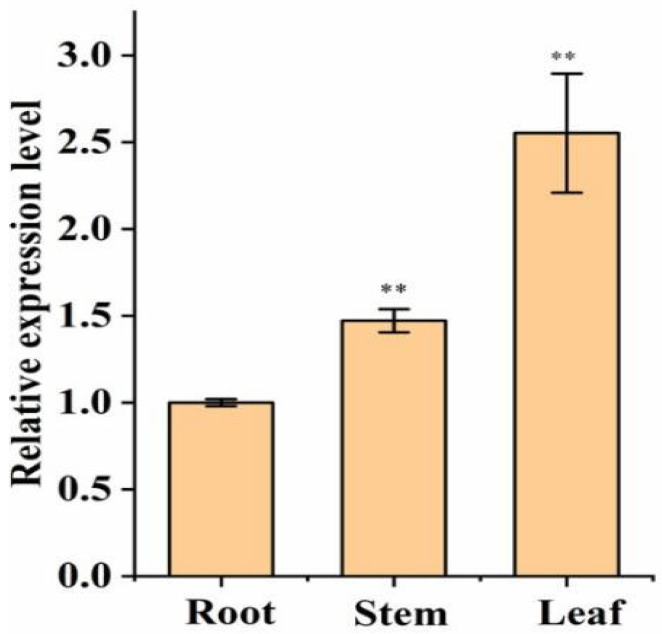
Tissue expression analysis of *Pid2of-MD33*. Values are the mean ± SD (*n* = 3; ** *p* < 0.01, *t*-test).

To investigate the tissue-specific expression of the *Pid2of-MD33* gene in *Oryza officinalis*, its expression levels in different tissues were analyzed using RT-qPCR. The results showed that *Pid2of-MD33* was expressed in various rice tissues, including roots, stems, and leaves, with the highest expression observed in the leaves (Figure 6). Furthermore, subcellular localization studies revealed that *Pid2of-MD33* predominantly functions at the plasma membrane.

### 2.8. Localization of PID2OF-MD33 Protein to the Plasma Membrane in Rice Cells

**Figure 7:**
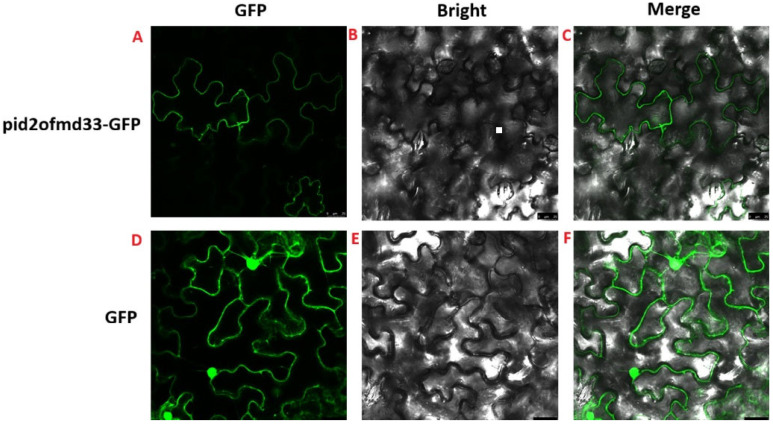
Subcellular localization of PID2OF-MD33. (**A**) PID2OF-MD33 under GFP green fluorescence; (**B**) PID2OF-MD33 under bright field view; (**C**) PID2OF-MD33 under compound field; (**D**) empty vector under GFP green fluorescence; (**E**) empty vector under bright field view; (**F**) empty vector under compound field.

The subcellular localization of *Pid2of-MD33* in tobacco leaves was investigated by constructing a *Pid2of-MD33*-GFP vector. An empty GFP vector served as a control. After transformation into *Agrobacterium* and infiltration into tobacco leaves, fluorescence microscopy revealed that PID2OF-MD33 was localized to the plasma membrane, whereas the GFP signal from the empty vector was evenly distributed throughout the cell. These results suggested that PID2OF-MD33 protein is targeted to the plasma membrane, indicating its potential biological function at this cellular site (Figure 7).

### 2.9. Construction of 35 s-pid2of-MD33 Overexpression Vector

**Figure 8:**
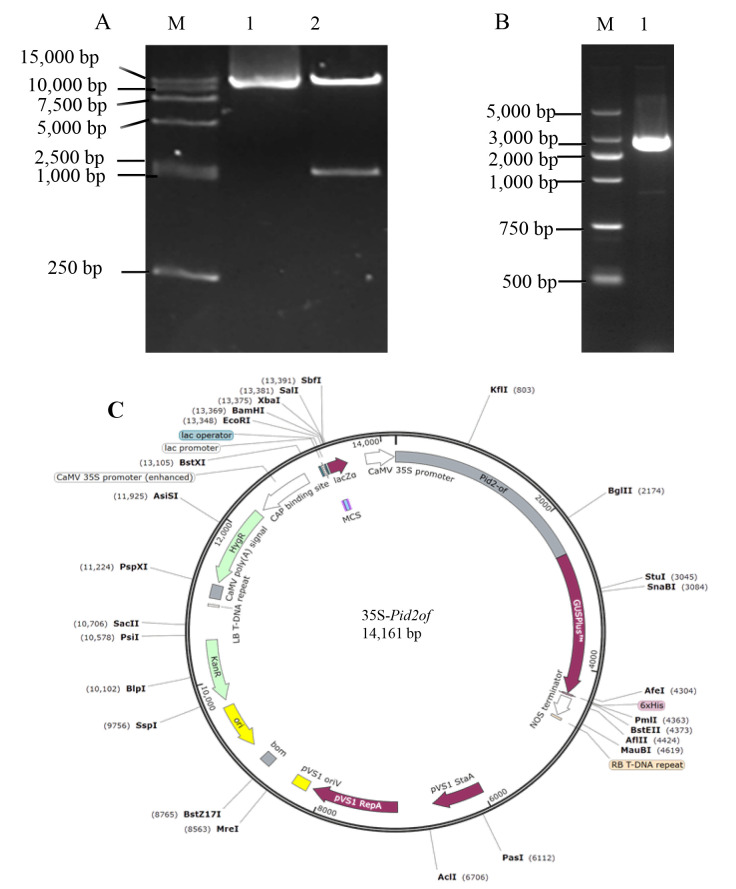
The electropherograms and vector map of 1305-*pid2of-MD33* construction. (**A**) Enzyme digestion of 35s-*pid2of-MD33*; M: DL15000 DNA Marker, 1: undigested recombinant 35s-*pid2of-MD33*; 2: restriction enzyme-digested recombinant plasmid 35s-*pid2of-MD33*; (**B**) cloning of *Pid2of-MD33*; 1: DL5000 DNA Marker; 2: the *Pid2of-MD33* gene; (**C**) the vector map of 35s-*pid2of-MD33*.

To generate the plant expression vector, the pCAMBIA1305 expression vector was double-digested with the restriction enzymes NcoI and SpeI to facilitate the insertion of the *pid2of-MD33* gene fragment. The target fragment was ligated into the linearized vector, and the recombinant plasmid was subsequently verified by double enzyme digestion, PCR, and DNA sequencing. A schematic representation of the enzyme digestion and PCR verification is shown in Figure 8A,B, confirming that the overexpression vector containing the *pid2of-MD33* gene was successfully constructed (Figure 8).

### 2.10. Molecular Screening and Verification of Transgenic Rice Lines Overexpressing Pid2of-MD33

**Figure 9:**
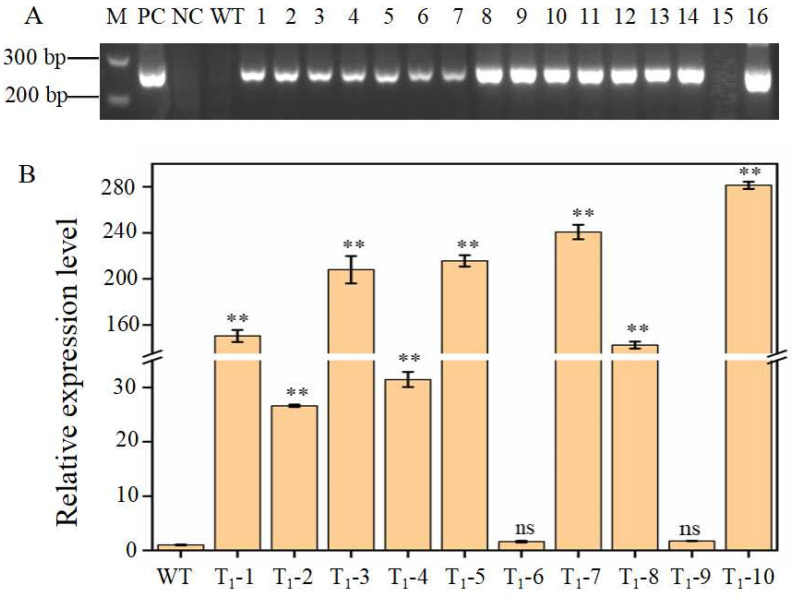
Positive identification of PCR and detection of *Pid2of-MD33* gene expression in overexpressed plants: (**A**) PCR identification of the T_1_ transgenic plants; M: DL1000 DNA Marker; PC: positive control; NC: negative control; WT: wild type; 1–16: T_1_ generation transgenic lines. (**B**) Relative expression levels of *pid2of-MD33* in T_1_ transgenic plants; WT: wild type; T_1_-1–T_1_-10: T_1_ generation transgenic positive plants; **: extremely significant differences; ns: not significant.

To verify the function of the *Pid2of-MD33* gene, an overexpression vector containing the CaMV35S promoter was constructed and introduced into the rice variety YG37 using *Agrobacterium*-mediated transformation. A total of 16 T_1_ generation transgenic lines were obtained. Positive transgenic plants were identified through PCR amplification of the Hygromycin resistance (hyg) gene and RT-qPCR analysis of *Pid2of-MD33* gene expression levels. PCR results showed that 15 out of the 16 T_1_ lines were positive for the *hyg* gene, with line 15 being the only negative. From the remaining 15 positive lines, 10 plants were randomly selected for expression analysis. RT-qPCR revealed that, except for plants 6 and 9, the expression of *pid2of-MD33* was significantly upregulated, ranging from 26-fold to 280-fold compared to the wild-type *O. officinalis*. These results confirmed that the selected plants were positive overexpression transgenic lines (Figure 9).

### 2.11. Rice Blast Resistance Phenotyping and Resistance Spectrum Analysis of Pid2of-MD33 Overexpressing Transgenic Plants

**Figure 10:**
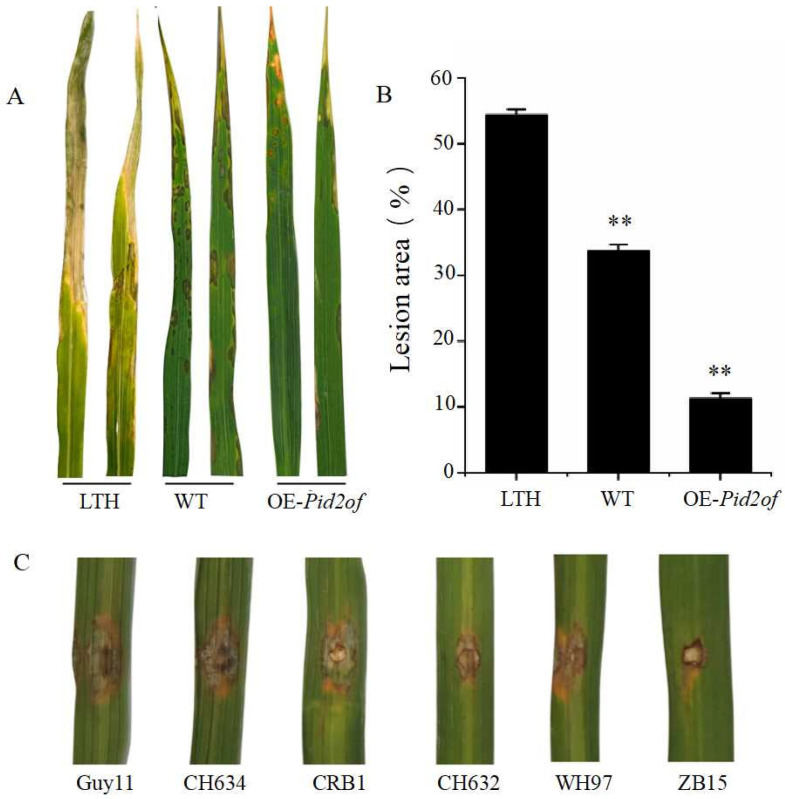
Identification of disease resistance phenotype of overexpressed lines: (**A**) disease resistance phenotype of different plants inoculated with ZB15; (**B**) lesion area after ZB15 inoculation (%); (**C**) resistance analysis of overexpressed lines to different *M. oryzae* strains; **: extremely significant difference.

In the rice variety YG37, which is susceptible to rice blast, the *pid2of-MD33* gene was overexpressed under the control of the strong CaMV35S promoter. After identifying positive overexpression lines, these transgenic plants, along with wild-type YG37 and the susceptible control LTH, were inoculated with the rice blast physiological race ZB15. Seven days after spray inoculation in a greenhouse, disease symptoms were assessed. As shown in Figure 10A, the overexpression lines exhibited significantly reduced lesion areas and fewer disease spots compared to wild-type YG37, indicating that *Pid2of-MD33* confers race-specific resistance to ZB15. To evaluate whether *Pid2of-MD33* also mediates broad-spectrum resistance, the positive overexpression lines were further inoculated ex vivo with additional rice blast physiological races: Guy11, CH634, CH632, WH97, and CRB1. As shown in Figure 10C, the gene conferred only limited resistance to these additional races. These results suggested that *pid2of-MD33* mediates specific resistance to ZB15, rather than broad-spectrum resistance.

### 2.12. H_2_O_2_ Accumulation in Rice Leaves 24 h Post-Inoculation with ZB15 Race

**Figure 11:**
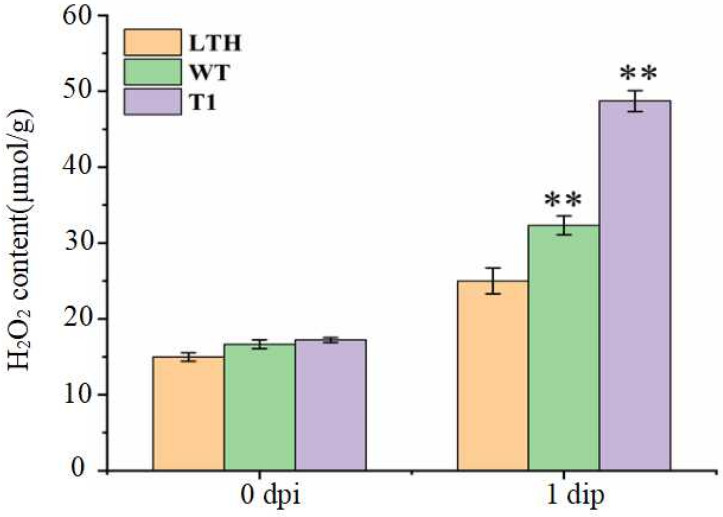
Determination of H_2_O_2_ content in different plants after inoculation. LTH: susceptible control; WT: wild type; T1: positive overexpressed plants, values are the mean ± SD (n = 3; ** *p* < 0.01 *t*-test).

Resistance to rice blast is often associated with changes in H_2_O_2_ levels within the plant [9]. To investigate the role of *pid2of-MD33* in modulating H_2_O_2_ accumulation during rice blast resistance, wild-type YG37, the susceptible control LTH, and positive overexpression lines at the three-leaf stage were spray-inoculated with *Magnaporthe oryzae* strain ZB15. Leaves from the same position were collected 24 h post-inoculation, and H_2_O_2_ content was measured spectrophotometrically. The results showed that H_2_O_2_ accumulation was low in both wild-type YG37 and susceptible control LTH (Figure 11). In contrast, the overexpression lines exhibited a significant increase in H_2_O_2_ levels following inoculation. These findings suggested that the *Pid2of-MD33* gene contributes to early defense responses in rice by promoting rapid H_2_O_2_ accumulation after pathogen challenge.

### 2.13. Gene Expression Analysis of Pathogenesis-Related Genes in Disease Resistance

**Figure 12:**
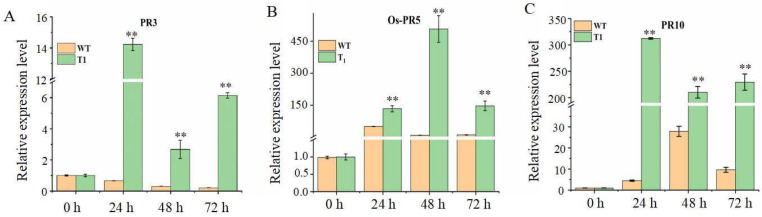
Expression level of PR gene;, values are the mean ± SD (*n* = 3; ** *p* < 0.01); (**A**): the expression level of OsPR3; (**B**): the expression level of OsPR5; (**C**): the expression level of OsPR10.

When *M. oryzae* (rice blast fungus) infects rice, it triggers an immune response that includes the rapid upregulation of pathogenesis-related (PR) genes, which play a key role in plant defense [23,24]. To assess the impact of *Pid2of-MD33* overexpression on defense gene activation, the expression levels of *OsPR3*, *OsPR5*, and *OsPR10* were analyzed in wild-type YG37 and positive overexpression lines at 0 h, 24 h, 48 h, and 72 h after inoculation with the *M. oryzae* physiological race ZB15. RT-PCR analysis revealed that the expression levels of all three PR genes were significantly upregulated in the overexpression lines at 24, 48, and 72 h post-inoculation compared to wild-type plants (Figure 12). These results indicate that *Pid2of-MD33* enhances the expression of key defense-related genes, contributing to the rice blast resistance phenotype.

## 3. Discussion

Rice blast, caused by the fungus *Magnaporthe oryzae* (syn. *Pyricularia oryzae*), is a major problem in rice cultivation and ranks among the most severe fungal diseases. Cloning and identifying resistance genes in rice, coupled with a comprehensive examination of the interaction between *M. oryzae* and rice, may provide insights into the mechanisms of rice disease resistance and facilitate the creation of new rice varieties with improved germplasm. These efforts are essential for protecting food security [7,15,25].

As the ancestral species of cultivated rice, wild rice has evolved remarkable stress-resistant traits through long-term adaptation to adverse environmental conditions in the wild [9,26]. Furthermore, it harbors a wealth of elite genetic resources. The utilization of disease-resistant genes from wild rice serves as the optimal approach to tackle challenges in current rice breeding, including the narrow genetic base and the impending exhaustion of superior varieties. Resistance breeding though has been the traditional objective of plant breeding programs. With changing scenarios, effective and diverse-resistant sources, particularly from wild relatives and from other sources, seem to be essential for the durability of resistance. Furthermore, precise tools are required for identification and transfer of genes for developing resistant genotypes [27,28,29]. To date, research on the exploration and utilization of resistance genes in *O. officinalis* has been predominantly focused on insect resistance, with more intensive investigations centered on resistance to brown planthoppers (BPH) and white-backed planthoppers (WBPH) [30,31]. The BPH-resistant and WBPH-resistant genes isolated from *O. officinalis* include *Bph11* [32], *Bph12* [33], *Bph14*, *Wbph7(t)*, and *Wbph8(t)* [34], among others. Nevertheless, studies on the exploration of blast resistance genes in *O. officinalis* remain scarce. Wild ancestors of cultivated rice are a natural genetic resource and contain a large number of excellent genes. *O. officinalis* belongs to the CC genome and is a well-known wild rice in south China [15,35]. In the present study, the function of the *Pid2of-MD33* gene, cloned from *O. officinalis* “Mengding Yaoye 33”, was validated through the construction of overexpression vectors and the generation of overexpressing transgenic plants. Following spray inoculation with the *M. oryzae* race ZB15, the overexpressing lines exhibited enhanced disease resistance phenotypes. However, resistance spectrum analysis revealed that this gene confers race-specific resistance exclusively to the ZB15 isolate. Cloned the *Pid2* allele from *Oryza rufipogon* in Yunnan Province and obtained transgenic lines with Nipponbare as the recipient material [25,36,37,38]. Characterization of the resistance spectrum of this allele demonstrated that it confers resistance to all 11 inoculated *M. oryzae* isolates [39]. A comparison with the results of the current study indicates that the *Pid2* allele from *O. rufipogon* in Yunnan Province possesses a broader resistance spectrum than the *Pid2* homolog from *O. officinalis* [23]. Nevertheless, the underlying mechanisms responsible for the differences in resistance spectra among homologous genes warrant further investigation. Analysis of the expression pattern of *Pid2of-MD33* showed no tissue specificity in its expression, with the highest transcript abundance detected in leaves, which is consistent with its role in leaf blast resistance. Subcellular localization results revealed that PID2OF-MD33 primarily exerts its biological functions at the plasma membrane, a distribution that corresponds to the functional role of RLK (receptor-like kinase) proteins in perceiving extracellular and intracellular signals and transducing them into the cell. Combined with the predictive analysis results of phosphorylation sites and interacting proteins of the encoded protein, it is hypothesized that this protein may respond to pathogen invasion via its extracellular domain, transmit signals into the cell, and regulate rice blast resistance through phosphorylation modification of downstream proteins [40,41,42]. In the overexpressing plants, the pathogenesis-related genes *OsPR3*, *OsPR5*, and *OsPR10* were activated upon inoculation with ZB15. This response constitutes a common event in plant innate immunity [43], indicating that PR genes are significantly regulated in the *Pid2of-MD33* mediated immune response against rice blast. However, the specific mechanisms by which these genes participate in the downstream immune response to ZB15 remain elusive and require in-depth subsequent investigations. Hydrogen peroxide (H_2_O_2_) plays diverse roles in plants, functioning crucially in signal transduction, participating in programmed cell death, and accumulating extensively under biotic or abiotic stress conditions [44]. In this study, H_2_O_2_ content was measured in overexpressing plants at 24 h post-inoculation with the pathogen, and it was found that H_2_O_2_ accumulation was significantly higher in overexpressing plants compared to the wild-type YG37. This finding suggests that the *Pid2of-MD33* gene triggers early defense responses in rice by promoting H_2_O_2_ accumulation. The elevated H_2_O_2_ accumulation was mainly attributed to the enhanced early defense responses and the intensified hypersensitive response (HR)-like oxidative burst triggered upon pathogen infection. Overexpression of the target gene accelerated the rapid production of reactive oxygen species, especially H_2_O_2_. As a crucial signaling molecule, H_2_O_2_ not only activates downstream defense pathways but also directly impedes pathogen invasion and spread by reinforcing cell wall reinforcement and restricting pathogen colonization. In addition, the increased H_2_O_2_ level may also be associated with altered activities of ROS-producing and ROS-scavenging systems, including NADPH oxidases, superoxide dismutase (SOD), catalase (CAT), and ascorbate peroxidase (APX), which collectively contribute to the higher overall ROS level in transgenic lines. Collectively, these changes contribute to the enhanced resistance of overexpression lines against *M. oryzae* strain ZB15.

A single amino acid substitution in the *Pid2* gene of cultivated rice gave rise to the susceptible allele *pid2* (441M), which is predominantly found in japonica rice. An intriguing question arises: did the susceptible allele emerge prior to the divergence of indica and japonica rice, or did it arise post-divergence? This study validated the blast resistance function of *Pid2of-MD33* from *O. officinalis*, which represents an “ancestral state” of the *Pid2* gene. This finding suggests that the susceptible allele *pid2* (441M) likely originated from a single nucleotide substitution in the resistant allele after the indica–japonica divergence. This evolutionary pattern appears distinct from that of blast resistance genes *Pita* and *Pik*, where resistant alleles are derived from susceptible ones [45]. Inoculation assays of *pid2of-MD33* overexpressing lines revealed a significant reduction in lesion area compared to the wild-type YG37, yet the plants were not completely immune to *M. oryzae* isolate ZB15. In contrast, ref. [46] reported that the *Pid2-Digu* genotype exhibited near-complete immunity to ZB15. Two key factors may account for this discrepancy. Firstly, divergent genetic backgrounds between experimental materials can modulate resistance phenotypes. Secondly, during the co-evolution of wild rice and its pathogens, genetic differentiation in wild rice populations likely drove corresponding pathogenicity diversification in *M. oryzae* strains. Wild rice-associated isolates generally exhibit weaker pathogenicity compared to those infecting cultivated rice [47,48,49]. It was hypothesized that the relatively mild resistance conferred by *Pid2of-MD33* reflects adaptation to less virulent pathogens in wild environments, whereas stronger resistance alleles in cultivated rice evolved in response to more aggressive pathogens. Previous pan-genome studies have demonstrated that wild rice possesses a significantly higher abundance and diversity of resistance-gene analogs (RGAs) compared with cultivated rice. Wild rice carries extensive genetic variation at resistance loci, and numerous alleles confer broad-spectrum and moderate resistance that is well adapted to the diverse, less virulent pathogen communities in natural ecosystems. In contrast, cultivated rice has experienced strong artificial selection on resistance loci during domestication, which has resulted in the enrichment of elite, high-intensity resistance alleles targeting specific, highly virulent pathogen strains in agricultural systems [49]. The domestication bottleneck has reduced resistance gene diversity in cultivars, promoting the fixation of specialized, strong resistance haplotypes that are effective against aggressive pathogens under intensive cultivation [18]. Collectively, these findings support a general evolutionary pattern in which wild rice retains diverse, mild resistance alleles adapted to natural pathogen pressures, whereas cultivated rice evolves robust, specialized resistance alleles under the selection of more aggressive pathogens in agroecosystems.

## 4. Materials and Methods

### 4.1. Plant Materials and Rice Blast Strains

Ten different populations of *O. officinalis* from Yunnan were used in the experiment during 2024 and 2025, including Jingneshanggou, Lancangmengkuang, Manlao, Manwo, Menghai, Mengzhe, Mengding28, Mengding33, Jinghong, and Kuanyexing populations. Additionally, the susceptible blast variety YG37 and LiJingXinTuanHeiGu (LTH) was included. The ZB15 blast strain was used as the main isolate for the detection of the *Pid2* gene. The Biotechnology and Genetic Resources Institute of Yunnan Academy of Agricultural Sciences provided this strain.

### 4.2. Genomic DNA Extraction and PCR Amplification

Genomic DNA was extracted from young leaf tissue using a modified cetyltrimethylammonium bromide (CTAB) method as described by [50]. The quantity and quality of the extracted DNA were assessed by 0.8% agarose gel electrophoresis and spectrophotometry using a NanoDrop ND-1000 (Thermo Fisher Scientific). DNA samples were then diluted with nuclease-free water to a final concentration of 20 ng/µL for PCR amplification. PCR was conducted in a 15 µL reaction volume, which included 2 µL of template DNA, 0.5 µM of each forward and reverse primer, 4.5 µL of Master Mix, and 7.5 µL H_2_O. The PCR reaction procedure was as follows: pre-denaturation at 94 °C for 5 min; denaturation at 94 °C for 30 s, annealing at 60 °C for 30 s, extension at 68 °C for 2.5 min, 35 cycles; extended at 68 °C for 10 min, incubated at 4 °C. The amplified PCR products were separated on 2–3% agarose gels stained with ethidium bromide. After electrophoresis and gel imaging, bands corresponding to the target fragment size, free of nonspecific products, were excised with a sterile scalpel. These gel bands containing the target DNA were then processed and kept using the Trelief^®^ DNA Gel Extraction Kit (Tsingke, Beijing, China) following the manufacturer’s instructions.

### 4.3. Transformation of Plasmid DNA into Competent Cells by Heat Shock Method

The transformation of plasmid DNA into bacteria using the heat shock method is an essential technique in molecular biology [51]. In this study, the pMD™18-T vector was used for transformation. *Escherichia coli DH5α* competent cells aliquoted into 100 μL per tube were taken out from the −80 °C ultra-low temperature refrigerator and immediately placed on ice for slow thawing for approximately 10 min until no ice crystals remained. After thawing, 8 μL (60 ng) of the plasmid to be transformed was added to the competent cells, and the tube bottom was flicked gently to mix thoroughly, followed by ice bath adsorption for 30 min. After incubation in ice, a mixture of competent bacteria and DNA was placed at 42 °C for 60 s (heat shock), not shaken, and then placed back in ice quickly (let stand for 2 min). Then, 700 μL of liquid LB was added without antibiotics, mixed well, and placed in a shaker at 37 °C, 200 rpm for 1 h. To assure isolating colonies, an appropriate amount of the solution obtained in the previous step was evenly spread on the LB solid medium containing the corresponding antibiotic and placed the inverted plate in the 37 °C incubator overnight. Monoclonal cells were picked into a centrifuge tube containing LB liquid medium containing 3~4 mL of corresponding antibiotics and incubated at 37 °C, 200 rpm overnight, and the obtained bacterial solution was sent to Sangon Bioengineering (Shanghai, China). For final confirmation, DNA sequencing was carried out, and the results were analyzed [52,53].

### 4.4. Bioinformatics Characterization of Pid2 Homologs Across O. officinalis Genotypes

According to the different genotype sequences of *Pid2of* homologous genes in *O. officinalis* obtained by cloning and sequencing, the gene sequences and other NCBIs in the tested *O. officinalis* and cultivated rice grains were analyzed by BioXM 2.7.1. Bioinformatics analyses were performed using MEGA7.0.26 software. Multiple sequence alignment was carried out with the MUSCLE algorithm, followed by manual trimming of non-conserved regions at both termini to ensure alignment quality. Phylogenetic trees were constructed using the Neighbor-Joining (NJ) method. The Poisson model was applied for amino acid sequences, while the Kimura 2-parameter model was used for nucleotide sequences. The reliability of branching was assessed by bootstrap analysis with 1000 replicates. Gaps were treated using the partial deletion method with a site coverage cutoff of 50%. Evolutionary distances among genes were also calculated with MEGA7 to analyze the evolutionary divergence characteristics of gene family members. A CD-search website (https://www.ncbi.nlm.nih.gov/Structure/cdd/wrpsb.cgi, accessed on 12 March 2026) was used to analyze the conserved functional domain and protein of the *Pid2of* gene type. ProtScale (https://web.expasy.org/protscale/, accessed on 7 April 2026) performed hydrophobic analysis of the encoded protein, NetPhos 3.1 Server (https://services.healthtech.dtu.dk/service.php?NetPhos-3.1 accessed on 5 June 2025) analysis of its phosphorylation site. SOPMA (https://npsa-pbil.ibcp.fr, accessed on 6 June 2025), and SWISS-MODEL (https://swissmodel.expasy.org/) predicted the secondary and tertiary structures of the encoded proteins; the Plant CARE website (https://bioinformatics.psb.ugent.be/webtools/plantcare/html/, accessed on 20 July 2025) analyzed their regulatory elements, and the STRING website (https://string-db.org/) predicted their interacting proteins.

### 4.5. Inoculation of Rice Blast Fungus

Conidia suspension of rice blast fungus was prepared using sterile water to gently scrape off the conidia on the solid medium of prune juice, trying not to scrape the medium, and then filtered through a magic filter cloth to remove the hyphae and other impurities. After that, we checked and observed the number of spores under the microscope and adjusted the concentration to 1.5~2 × 10^5^ spores mL^−1^ as described by [54]. For in vitro inoculation, 1 mg/L of 6-Benzyladenine aqueous solution was configured and poured into a Petri dish. Then, we cut rice leaves of materials of the same length and used a 10 μL pipette tip to gently poke a small wound on the leaf to ensure that the leaves are not punctured. After that, the treated leaves were put into a Petri dish filled with 6-BA, absorbed 10 μL of the prepared spore suspension dropped into the wound, and were gently transferred to the incubator. For the first 24 h, the leaves were cultured in the dark, then in 12 h of light, and 12 h of darkness. The temperature was 28 °C; the humidity was 80%. After 7 days, the lengths of the lesions were investigated.

For spray inoculation, rice seedlings at the 3rd–4th leaf stage from transgenic plants were first placed with susceptible controls and wild-type rice in a greenhouse inoculation box (we poured an appropriate amount of water at the bottom of the box in advance to moisturize the box). Then, we sprayed rice leaves in the inoculation box with a rice blast spore suspension. After that, the whole inoculation box was wrapped with plastic wrap and black cloth cover. The experiment was carried out under controlled growth conditions at 28 °C and 90% relative humidity for an initial 24 h. After 24 h, the black cloth cover was removed. Then, we shifted to 16/8 h light/dark regime. The disease reaction observed in each rice line was recorded after 7 days post-inoculation using a 0–5-point disease rating scale [8].

### 4.6. Determination of Hydrogen Peroxide Content

The leaves of the samples were cut 24 h after inoculation to detect the hydrogen peroxide content (H_2_O_2_) by spectrophotometry. Hydrogen peroxide (H_2_O_2_) content was determined using a commercial assay kit (Solarbio, Beijing, China) based on the titanium sulfate colorimetric method. Briefly, approximately 0.1 g of sample leaves were harvested and cut 24 h post-inoculation, then weighed and homogenized in pre-cooled acetone on ice. The homogenate was centrifuged at 8000× *g* for 10 min at 4 °C, and the supernatant was collected and kept on ice for subsequent detection. A standard curve was prepared by diluting the 1 mmol/mL H_2_O_2_ standard solution (included in the kit) with acetone to a series of concentrations (0, 0.01, 0.05, 0.1, 0.5, and 1 μmol/mL). For the colorimetric reaction, 250 μL of sample supernatant, standard solution, or acetone (as the blank control) was added to separate EP tubes, followed by 25 μL of titanium sulfate reagent (reagent II, dissolved in concentrated hydrochloric acid before use) and 50 μL of reaction buffer (reagent III). After thorough mixing, the tubes were centrifuged at 4000× *g* for 10 min at room temperature, and the supernatant was discarded. The resulting precipitate was washed 3–5 times with acetone to remove plant pigments, and then 250 μL of strong acid dissolving solution (reagent IV) was added to dissolve the precipitate, followed by incubation at room temperature for 5 min. Finally, 200 μL of the reaction solution was transferred to a microplate, and the absorbance was measured at 415 nm using a spectrophotometer or microplate reader, with the blank tube used for zero adjustment. The H_2_O_2_ concentration in the samples was calculated according to the standard curve plotted with the absorbance values and known concentrations of the H_2_O_2_ standard solution, and the final content was expressed as μmol/g fresh weight.

### 4.7. RT-qPCR Analysis and Gene Expression Levels

Total RNA was extracted from fresh plant tissues using the FastPure^®^ Universal Plant Total RNA Isolation Kit (Vazyme, Nanjing, China) following the manufacturer’s instructions. Briefly, approximately 0.1 g of sample leaves was harvested at 0 h, 12 h, 24 h, 48 h and 72 h after inoculation and ground into a fine powder in liquid nitrogen. The powder was then resuspended in 400 μL of Lysis Buffer (supplied in the kit) and vortexed vigorously to ensure complete homogenization. After incubation at room temperature for 5 min, the lysate was centrifuged at 12,000× *g* for 2 min to remove cell debris. The clear supernatant was transferred to a new column, and 80 μL of Buffer RPE was added to precipitate impurities. Following centrifugation at 12,000× *g* for 2 min, the supernatant was transferred to a new collection tube, and an equal volume of absolute ethanol was added and mixed thoroughly. The mixture was then loaded onto an adsorption column and centrifuged to allow RNA binding. The column was washed twice with 500 μL of Wash Buffer (supplied in the kit) to remove contaminants. Finally, the purified RNA was eluted with 30–50 μL of RNase-free water, and the concentration and purity were determined using a NanoDrop spectrophotometer.

Quantitative real-time reverse transcription PCR (*RT-qPCR*) was performed using the KAPA SYBR FAST *RT-qPCR* Master mix (Kapa Biosystems, Inc., Wimington, MA, USA). The experiment was carried out following the manufacturer’s instructions with slight modifications, and all operations were performed on ice to prevent RNA degradation and ensure reaction stability. Briefly, the qRT-PCR reaction system (20 μL total volume) was prepared as follows: 10 μL of 2× KAPA SYBR FAST *RT-qPCR* Master mix, 0.4 μL of upstream primer (10 μmol/L), 0.4 μL of downstream primer (10 μmol/L), 2 μL of cDNA template (diluted to an appropriate concentration according to the RNA concentration), and 7.2 μL of RNase-free water. After gentle mixing, the reaction mixture was centrifuged briefly to collect the liquid at the bottom of the tube, avoiding bubbles that might affect fluorescence detection. The RT-qPCRwas performed on an real-time quantitative PCR (qPCR) instrument using the LightCycler 480 II PCR System (Roche) [55].

### 4.8. Analysis of Pathogenesis-Related Gene Expression Levels

For *RT-qPCR* analysis, Actin was used as the internal reference gene. Total RNA was isolated from wild-type YG37 and positive overexpression lines at 0 h, 24 h, 48 h, and 72 h post-inoculation with *M. oryzae* strain ZB15 using the Spectrum Plant Total RNA Kit (Sigma-Aldrich, St. louis, MO, USA) and was subsequently reverse-transcribed into cDNA. Using the resulting cDNA as the template, qPCR amplification was performed with gene-specific primers for the pathogenesis-related genes *OsPR3*, *OsPR5*, and *OsPR10*. The reaction mixture and thermal cycling conditions were consistent with those described in Section 4.2, and the expression levels of *OsPR3*, *OsPR5*, and *OsPR10* were accordingly analyzed.

## 5. Conclusions

This study uncovers novel gene resources relevant to the interaction between rice and *M. oryzae*, the causal agent of rice blast disease. Additionally, it establishes a theoretical framework for utilizing *O. officinalis* in rice blast resistance breeding in the future. Furthermore, this research lays a foundational understanding of the resistance mechanisms associated with *Pid2* homologous genes in wild rice, opening new avenues for developing more effective and resilient rice varieties.

## Figures and Tables

**Table 1:** Genotypes of *Pid2* in different *O. officinalis* populations.

Genotype			Varied Locus in ORF of *Pid2of*						
	105	124	327	829	1171	1228	1944	2043	2229
JH	T	A	C	G	C	G	A	G	A
MD28	T	A	C	G	A	A	A	C	A
MD33	A	G	T	T	C	G	T	G	C

**Table 2:** Polymorphism analysis and neutrality test of *Pid2* and its alleles.

Group	Region	Parsimony Informative Sites	SNPs	π	θw	Tajima’s D	K
All	ORF	116	123	0.02653	0.01871	2.21564 *	67.250
	B-lectin	18	19	0.03004	0.02118	2.17159 *	10.393
	PAN	24	25	0.05378	0.03723	2.33960 **	13.929
	PKc-like	21	22	0.01541	0.01058	1.91290	12.107
*O. officinalis*	B-lectin	1	2	0.00337	0.00315	0.59158	1.167
	PAN	0	2	0.00386	0.00421	−0.70990	1.000
	PKc-like	2	4	0.00291	0.00272	0.65010	2.333
*O. sativa*	B-lectin	0	0	0	0	0	0
	PAN	0	0	0	0	0	0
	PKc-like	0	1	0.00062	0.00068	−0.61237	0.500

Note: * *p* < 0.05, ** *p* < 0.01.

**Table 3:** Analysis of promoter elements of *Pid2of-MD33*.

Element	Sequences	Number	Function
chs-CMA1a	TTACTTAA	1	part of a light-responsive element
I-box	TGATAATGT	1	part of a light-responsive element
GT1-motif	GGTTAA	2	light-responsive element
TCA-element	CCATCTTTTT	2	cis-acting element involved in salicylic acid responsiveness
LS7	CAGATTTATTTT TA	1	part of a light-responsive element
CGTCA-motif	CGTCA	1	cis-acting regulatory element involved in the MeJA-responsiveness
TGACG-motif	TGACG	1	cis-acting regulatory element involved in the MeJA-responsiveness
AE-box	AGAAACAA	3	part of a module for light response
ARE	AAACCA	3	cis-acting regulatory element essential for the aerobic induction
P-box	CCTTTTG	3	gibberellin-responsive element
MBS	CAACTG	2	MYB binding site involved in drought inducibility

## Data Availability

The original contributions presented in this study are included in the article. Further inquiries can be directed to the corresponding authors.

## Abbreviations

The following abbreviations are used in this manuscript:

*O. officinalis*	*Oryza officinalis*
*M. oryzae*	*Magnaporthe oryzae*
DNA	Deoxyribonucleic Acid
CTAB	Cetyltrimethylammonium Bromide
PCR	Polymerase Chain Reaction
LB	Luria–Bertani medium
6-BA	6-benzylaminopurine
RH	Relative Humidity
H_2_O_2_	Hydrogen Peroxide
ORF	Open Reading Frame
SNPs	Single Nucleotide Polymorphisms
